# Anxious Behavior of Adult CD1 Mice Perinatally Exposed to Low Concentrations of Ethanol Correlates With Morphological Changes in Cingulate Cortex and Amygdala

**DOI:** 10.3389/fnbeh.2020.00092

**Published:** 2020-06-19

**Authors:** Catalina Madarnas, Nerina Mariel Villalba, Delia Soriano, Alicia Brusco

**Affiliations:** ^1^Instituto de Biología Celular y Neurociencia (IBCN), Universidad de Buenos Aires, CONICET, Buenos Aires, Argentina; ^2^Facultad de Medicina, Departamento de Biología Celular, Histología, Embriología y Genética, Universidad de Buenos Aires, Buenos Aires, Argentina

**Keywords:** perinatal, ethanol, cingulate cortex, amygdala, behavior, adult, anxious behavior

## Abstract

Perinatal ethanol (EtOH) exposure is associated with high incidence of behavioral disorders such as depression and anxiety. The cerebral areas related with these consequences involve the corticolimbic system, in particular the prefrontal cortex, hippocampus, amygdala, and cingulate cortex, although the latter has not been thoroughly studied yet. Different animal models of prenatal or perinatal EtOH exposure have reported morphofunctional alterations in the central nervous system, which could explain behavioral disorders along life; these results focus on youth and adolescents and are still controversial. In the light of these inconclusive results, the aim of this work was to analyze adult behavior in CD1 mice perinatally exposed to low concentrations of EtOH (PEE) during gestation and lactation, and describe the morphology of the cingulate cortex and amygdala with a view to establishing structure/function/behavior correlations. Primiparous CD1 female mice were exposed to EtOH 6% v/v for 20 days prior to mating and continued drinking EtOH 6% v/v during pregnancy and lactation. After weaning, male pups were fed food and water *ad libitum* until 77 days of age, when behavioral and morphological studies were performed. Mouse behavior was analyzed through light–dark box and open field tests. Parameters related to anxious behavior and locomotor activity revealed anxiogenic behavior in PEE mice. After behavioral studies, mice were perfused and neurons, axons, serotonin transporter, 5HT, CB1 receptor (CB1R) and 5HT1A receptor (5HT1AR) were studied by immunofluorescence and immunohistochemistry in brain sections containing cingulate cortex and amygdala. Cingulate cortex and amygdala cytoarchitecture were preserved in adult PEE mice, although a smaller number of neurons was detected in the amygdala. Cingulate cortex axons demonstrated disorganized radial distribution and reduced area. Serotonergic and endocannabinoid systems, both involved in anxious behavior, showed differential expression. Serotonergic afferents were lower in both brain areas of PEE animals, while 5HT1AR expression was lower in the cingulate cortex and higher in the amygdala. The expression of CB1R was lower only in the amygdala. In sum, EtOH exposure during early brain development induces morphological changes in structures of the limbic system and its neuromodulation, which persist into adulthood and may be responsible for anxious behavior.

## Introduction

Maternal alcohol consumption produces a spectrum of deleterious effects on offspring whose incidence is around 10% in the general population (Popova et al., 2017). Fetal alcohol spectrum disorders (FASD) encompass a range of pathological conditions resulting from alcohol consumption of different magnitudes and during different stages of pregnancy (Riley and McGee, 2005; Hoyme et al., 2016), which include cognitive, behavioral, and adaptive functional deficits (Mattson et al., 2019). Attention has focused on FASD as a serious public health issue and has encouraged research into the basic mechanisms of prenatal alcohol exposure and its long-term consequences (Koren and Navioz, 2003).

As part of FASD, the fetal alcohol syndrome (FAS) represents one of the most severe conditions. Several studies have examined the extent to which the frequency and severity of FAS are related to the amount of alcohol consumed and the temporal pattern of consumption (Sayal et al., 2009; May et al., 2011, 2013). Ethanol (EtOH) exposure during pregnancy has been then found to cause serious morphological, behavioral, and cognitive alterations in developing children, which may also persist into adulthood (Mattson et al., 2001; Lebel et al., 2011; Donald et al., 2016; Hoyme et al., 2016). Neurobehavioral impairment has been documented not only in FAS children severely exposed to EtOH but also in children prenatally exposed to moderate EtOH doses (O’Connor and Paley, 2006; Murray et al., 2016). Moreover, alcohol abuse prior to pregnancy may have persistent adverse effects that are not obliterated by abstinence during pregnancy.

Studies in both humans and animals have extensively demonstrated the deleterious effects of maternal alcohol ingestion on the fetus (Aronne et al., 2008, 2011; Gil-Mohapel et al., 2010; Ornoy and Ergaz, 2010). In individuals affected by FASD, secondary alterations have been described, which mainly include mental health disorders (Barr et al., 2006; Weyrauch et al., 2017). Clinical studies suggest a correlation between prenatal EtOH exposure and the incidence of anxiety-related disorders during adolescence and adulthood (Barr et al., 2006). Additionally, it has also been reported that individuals with FASD present structural and functional alterations in different areas of the brain, among which structures belonging to the limbic system stand out (Mattson et al., 2001; Nardelli et al., 2011; Malisza et al., 2012; Roussotte et al., 2012; Yang et al., 2012; Wozniak et al., 2013; Donald et al., 2016). This system is involved in emotional processing and is formed by structures such as the amygdala, cingulate cortex, prefrontal cortex, and insula (Davidson et al., 2003). Alterations in these areas of the brain have been associated with numerous behavioral disorders, such as depression and anxiety (Aggleton and Brown, 1999; Dalgleish, 2004).

The effects of maternal alcoholism on offspring behavior have been widely studied in animal models, although the results reported are controversial and focus mainly on the periods of childhood and adolescence. Some authors have reported locomotor hyperactivity as one of the most characteristic effects of *in utero* EtOH intoxication, while others have found no hyperactivity (Abel and Berman, 1994; Tran et al., 2000; Downing et al., 2008; Brys et al., 2014).

It has also been observed that early exposure to alcohol—passive or in the context of operant learning schemes—alters consumption evaluated at later stages of development (Spear and Molina, 2005). Furthermore, a growing number of studies using rodents consistently demonstrate that prenatal EtOH exposure induces increased postnatal EtOH intake, as observed in studies in which EtOH was administered to the pregnant dam during most of gestation (Arias and Chotro, 2005; Youngentob et al., 2007; Aronne et al., 2013; Brancato et al., 2018).

The literature is also particularly controversial about anxiety-like behavior. While some authors have recorded an anxiogenic effect of *in utero* EtOH exposure (Hellemans et al., 2008; Cullen et al., 2013; Wille-Bille et al., 2018), others have reported a decrease in anxiety, even using similar animal treatments and models (Carneiro et al., 2005; Ohta et al., 2010; Diaz et al., 2016).

Indeed, a systematic review has recently shown the limited evidence available in the literature on the association between fetal alcohol exposure and offspring emotional problems in childhood or adolescence, in particular anxiety and depression (Easey et al., 2019). In the same way, animal model studies on adult offspring prenatally exposed to EtOH do not abound.

In this context, the aim of the present work was to determine the impact of alcohol exposure by analyzing animal behavior and the morphology of cingulate cortex and amygdala, two brain areas related to emotional behavior, in adult CD1 mice perinatally exposed to EtOH at low concentrations.

## Materials and Methods

### Animals and Animal Care

All procedures were in agreement with standards for the care of laboratory animals as outlined in the National Institutes of Health Guide for the Care and Use of Laboratory Animals. All procedures were administered under the auspices of CICUAL, Facultad de Medicina, Universidad de Buenos Aires (Res. CD 2375/2017).

Twelve CD1 primiparous females (aged 45–50 days) and six adult CD1 males, all provided by the animal room at the Institute of Cell Biology and Neuroscience, were housed in cages (two females per cage and three males per cage) in a temperature (22–23°C) and photoperiod (12-h light/dark)-controlled room, with lights on between 08:00 and 20:00 h. Both the 12 females and the six males were randomly selected from different litters of the CD1 colony.

### EtOH Exposure

**Figure 1:**
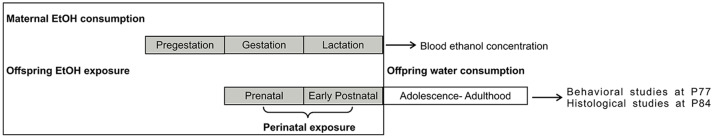
Experimental procedure. E mothers consumed 6% v/v ethanol (EtOH) as the only beverage *ad libitum* during pregestational, gestational, and lactation periods. PEE pups were thus exposed to EtOH from conception to weaning, after which they drank water as the only beverage and had no further contact with EtOH. P, postnatal day.

Female mice were divided into two groups, a control group (C, six females) and an EtOH-exposed group (E, six females), and housed two in each cage. As from 20 days before mating, E female mice received a constant dilution of 6% v/v EtOH in water as the only beverage with standard food *ad libitum* until pup weaning. C female mice and all male mice received water and standard food *ad libitum*. One male mouse was put in each cage for mating, and pregnancy was determined by the detection of a vaginal plug (considered gestational day 0). Pregnant mice were separated, one per cage, for the rest of pregnancy and nursing. At postnatal day 1 (P1), all the litters were reduced to no more than 10 pups, preferentially male, to be used for the different studies. At P21, male offspring were separated from their mother and housed 3 to 6 per litter in each cage, with water and standard food *ad libitum* and no further contact with EtOH. Pups from E mothers were defined as the perinatally exposed to EtOH group (PEE) and the pups from C mothers the Control group. E mothers and PEE females were used for blood EtOH concentration (BEC) measurements at the end of lactation. C mothers and Control female pups were returned to complete their life as part of the colony, and male offspring of the two groups (Control and PEE) were submitted to behavioral studies at P77 and subsequently perfused for morphological measurements (Figure 1).

Dams’ weight gain and beverage intake were controlled during pregestational, gestational, and lactation period. Once the litters were born, the number of offspring was counted and the pups’ appearance was qualitatively evaluated. Maternal care behavior was qualitatively assessed three times a week during lactation period, which included observation of nest building, group care—not separating pups from the others—appropriated pups nursing, retrieving the pups to the nest when they were moved in cage changing, and being in contact with the pups. Also, male pups’ body weight was registered at P21 and adulthood.

#### Blood EtOH Concentration

Post-weaning E dams and P21 PEE females were anesthetized to obtain blood samples from carotid arteries and later euthanized. The blood samples were collected in the light cycle, between 09:00 and 3:00 h, that is, 1–4 h after lights turn on according to authors who analyzed the peak of BEC in rodents (Simpson et al., 2005; Juarez et al., 2017). Both dam and P21 offspring BEC was determined in a spectrophotometer by means of an enzymatic method with a specific Quanti Chrom EtOH Assay Kit (Bioassay Systems).

### Behavioral Studies of Adult CD1 Mice Perinatally Exposed to EtOH

Between P77 and P84, male PEE and Control pups corresponding to six different Control and PEE litters (see Supplementary Table S1) were tested for anxiety and locomotor activity in a behavioral test battery including the light–dark box test (LDB) followed by the open field test (OF). These two tests were conducted 1 week from one another and all animals performed the two test batteries in the specific order mentioned.

All tests were performed between 9:00 am and 2:00 pm, and animals were taken to the test room the day before at 5:00–6:00 pm. Once the three tests had finished, animals were returned to their housing room until morphological analyses.

#### LDB

The device used consists of two compartments (20 cm high, 20 cm wide, 15 cm deep) connected by a hole (4 cm wide and 5 cm high). Animals were placed in the light compartment, facing the hole. Animals were filmed for later video analysis and time spent in the light compartment and time spent in the dark compartment, and the number of transitions was registered during the next 5 min.

#### OF

The apparatus (50 cm wide, 50 cm long, 40 cm high) consists of an area with black plywood walls and wooden floor, divided into 16 squares by white lines (four central, 12 peripheral). The animals were put on the central area and were recorded with a video camera for 5 min for later analysis. Time spent in the central area, time in the peripheral area, latency, total distance traveled, and number of rearings and thigmotaxis were measured.

### Morphological Studies

After the behavioral test battery, 10 male mice per experimental group were randomly selected from all litters and deeply anesthetized with ketamine and xylazine in doses of 100 and 10 mg/kg, respectively. Animals were then perfused through the left ventricle, initially with physiological solution added to 50 IU heparin, and subsequently with a fixative solution containing 4% (w/v) paraformaldehyde in 0.1 M phosphate buffer (PB), pH 7.4. Brains were removed and postfixed in the same cold fixative solution for 4 h. Brains were then washed overnight in 5% (w/v) sucrose in PB at 4°C. Afterwards, brains were cryoprotected by immersion in a solution containing 30% (w/v) sucrose in PB and stored at −80°C until used. Coronal 50-μm-thick brain sections were cut using a cryostat (Leitz, Kryostat 1720 Digital), put in Eppendorf vials containing glycerol 50% in phosphate buffer saline (PBS), and stored at −20°C until used, or cut into 20-μm-thick brain sections and mounted directly on gelatin-coated slides. Brain sections corresponding to 1.10–0.02 mm Bregma level for anterior cingulate cortex (ACC) and −1 to −2.30 mm Bregma level for amygdala (Franklin and Paxinos, 2008) were processed for the corresponding histological studies.

#### Immunofluorescence

Five coronal 50-μm-thick brain sections containing ACC and amygdala were randomly selected from five mice per group, each one from different litters. Slices were washed three times in PBS and immersed in a solution of 3% (v/v) normal equine serum plus 0.5% (v/v) Triton X-100 in PBS for 3 h at 4°C under agitation to permeabilize and block unspecific sites. Sections were then incubated with the following primary antibodies diluted in a solution of 1% (v/v) normal equine serum and 0.3% (v/v) Triton X-100 in PBS: mouse anti-NeuN (mouse anti-neuronal nuclei, monoclonal antibody, 1:1,000, Millipore, Cat# MAB377, RRID:AB_2298772), rabbit anti-5HT1A receptor (5HT1AR; rabbit anti-serotonin receptor type 1A, polyclonal antibody, 1:1,000, Millipore, Cat# AB15350, RRID:AB_805421), rabbit anti-CB1 receptor (CB1R, rabbit anti-cannabinoid receptor type 1, 1:3,000, Cayman Chemicals, Cat# 10006590, RRID:AB_10098690), rabbit anti-5HT (rabbit anti serotonin, polyclonal antibody, 1:1,000, developed in our laboratory; Brusco et al., 1983), and mouse anti-5HTT (mouse anti-serotonin transporter, monoclonal antibody 1:1,000, Millipore, Cat# MAB1564, RRID:AB_94220). Slices were incubated at 4°C overnight under agitation. After three washes in PBS, sections were incubated for 1.5 h in the dark with fluorescent secondary antibodies: goat anti-mouse IgG conjugated with Alexa Fluor^TM^ 568 (1:1,000, Invitrogen, Cat# A11004, RRID:AB_143162) and goat anti-rabbit IgG conjugated with Alexa Fluor^TM^ 488 (1:1,000, Invitrogen, Cat# A11008, RRID:AB_143165). In each immunofluorescence study, a negative control was performed omitting the primary antibody to ensure technique specificity (see Supplementary Figure S2). Sections were later counterstained with Hoechst 33342 (1:1,000, Sigma-Aldrich) to label nuclei, mounted on gelatin-coated slides, and coverslipped with 70% glycerol mounting medium.

Photographs were taken in an inverted Olympus IX83 microscope with several objectives (4×, 10×, 20×). For double immunofluorescence studies, an objective of 60× and an additional spinning disk unit (SDU) for better resolution were used to analyze two markers in a brain area and to show marker colocalization. Images were acquired using high-resolution digital monochromatic sCMOS *Orca* camera (Hamamatsu) and *CellSens Dimension CS-DI-V1* software.

#### Immunoperoxidase

Five coronal 50-μm-thick brain sections containing ACC and amygdala were randomly selected from five mice per group, each one from different litters. Slices were washed three times in PBS and immersed in a solution of 0.5% (v/v) H_2_O_2_ in PBS for 1 h at room temperature under agitation to inhibit endogen peroxidase. Sections were then washed three times in PBS and immersed in a solution of 3% (v/v) normal equine serum plus 0.5% (v/v) Triton X-100 in PBS for 1.5 h at room temperature under agitation to permeabilize and block unspecific sites. Sections were then incubated with the following primary antibodies diluted in a solution of 1% (v/v) normal equine serum and 0.3% (v/v) Triton X-100 in PBS: mouse anti-MAP2 (mouse anti-microtubule-associated protein type 2, monoclonal antibody, 1:1,000, Sigma-Aldrich, Cat# M4403, RRID:AB_477193) and mouse anti-NF200 (mouse anti-neurofilament 200 kDa, monoclonal antibody, 1:1,000, Sigma-Aldrich, Cat# N0142, RRID:AB_477257). Slices were incubated at 4°C overnight under agitation. After three washes in PBS, sections were incubated for 1.5 h with goat anti-mouse IgG biotin conjugated (whole molecule, polyclonal antibody, 1:1,000, Sigma-Aldrich Cat# B7264, RRID:AB_258607). After three washes in PBS, sections were incubated with extravidin peroxidase solution (1:500, Sigma-Aldrich, Cat# E2886) followed by two washes in PBS and two with acetate buffer (AB) 0.1 M pH 6. Slices were incubated with 0.035% (w/v) 3,3′ diaminobenzidine (Sigma Aldrich) and 4% (w/v) nickel ammonium sulfate in AB, added H_2_O_2_ to reveal color, then washed twice with AB, and finally washed with distilled water. Slices were mounted on gelatin-coated slides and coverslipped using Canada Synthetic Balm as mounting media.

Photographs were taken on a Zeiss Axiolab microscope with several objectives (2.5×, 10×, 20×). Images were acquired using CCD *Q-Color 3* camera (Olympus) and *QCapture 6.0* software.

#### Morphometric Digital Image Analysis

All measurements were made on the photomicrographs taken with the corresponding microscopes and analyzed by two blinded operators. ACC and amygdala were the two brain areas selected for morphometric studies, and all the measurements were made using ImageJ software (NIH^1^).

From immunostaining, the number of neuron nuclei per unit of area, the percentage of area covered by 5HTT, 5HT, NF200, and MAP2-positive fibers as well as the percentage of area covered by 5HT1AR and CB1R-positive immunostained structures were measured in 20× primary magnification images. The number of cells per unit of area was determined by quantification of Hoechst-positive nuclei at 20× primary magnification. The percentage of area covered by immunolabeled fibers or receptor was related to the total area of the corresponding microscopic field at 20× primary magnification.

To measure the level of dispersion of the directionality of NF200-positive fibers and MAP2-positive dendrite orientation, ACC photomicrographs for each marker were analyzed with ImageJ. The Directionality plugin was used following instructions on https://imagej.net/Directionality, which exploits the local gradient orientation method (Schindelin et al., 2012; Schneider et al., 2012) for this quantification. For more information about quantification with this plugin, see Supplementary Material Section 3.

To measure 5HT and 5HTT colocalization in ACC and amygdala, photomicrographs of double-immunostaining with both markers were analyzed with ImageJ, using the JACoP plugin. The threshold from which it was considered a positive mark was set for each marker and the Manders’ overlap coefficient was calculated (Manders et al., 1992; Bolte and Cordelières, 2006).

### Data Analysis

Statistical analysis was performed using GraphPad Prism v5.00 (GraphPad Software Inc.). In behavioral tests, an average for each litter from both experimental groups was calculated (see Supplementary Table S1). A statistical Student’s *t*-test was performed to compare the means of the averages per litter of Control and PEE groups for all parameters. Model assumptions were verified in all cases.

Immunostaining quantifications (*n* = 3–5/treatment) were made from three slices per brain of each treatment and brain structure. Means and standard error of the mean (SEM) were obtained for all variables measured, the assumptions of normality and homoscedasticity were tested, and a two-tailed Student’s *t*-test was performed to compare the two groups.

## Results

### Physical Parameters of PEE Mice

Neither microcephaly nor any physical malformation was observed in PEE pups. There were neither litter size variations (Control 13.83 ± 0.4773 *n* = 6 vs. PEE 13.17 ± 0.7923 *n* = 6, Student’s *t*-test, ns) nor weight deviations in P21 (Control 12.75 g ± 0.4787 *n* = 4 vs. PEE 12.50 g ± 0.2887 *n* = 4, Student’s *t*-test, ns) and adulthood (Control 34.13 ± 0.6152 *n* = 32 vs. PEE 35.32 ± 0.4962 *n* = 28, Student’s *t*-test, ns) between PEE and Control pups.

There were no differences observed between the behavior of C and E mothers regarding the maternal care parameters qualitatively assessed. Ethanol mothers’ consumption during pregestational, gestational, and lactation periods is shown in Supplementary Figure S1. There were no differences between E and C mothers in weight gain and beverage intake during pregestational, gestational, and lactation periods (Supplementary Figure S1). E mothers yielded BEC values of 73.29 ± 8.69 mg/dl (*n* = 3) at the end of lactation, while female PEE pups yielded a BEC of 101.56 ± 5.21 mg/dl (*n* = 2) at P21.

### Behavioral Studies

#### LDB

**Figure 2:**
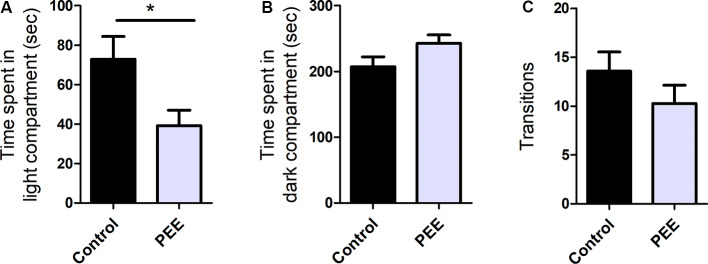
Perinatal EtOH exposure increases anxiety responses in the light-dark box test (LDB) test. Time spent in light compartment (in seconds, **A**), time spent in dark compartment (in seconds, **B**), number of transitions between the two compartments **(C)**. Data expressed as the mean ± standard error of the mean (SEM); all parameters were analyzed by Student’s *t*-test (*p < 0.05). Control *n* = 6; PEE *n* = 6, each data correspond to the average per litter.

PEE mice showed significant differences with respect to Control ones, spending less time in the light compartment (*t*_(10)_ = 2.414, *p* = 0.0364; Figure 2A), which indicates anxiety-like behavior. Although no significant differences were observed, it can be noticed that PEE males tend to spend more time in dark compartment compared to Controls (Figure 2B), which also suggests an increase in anxiety. In turn, the number of transitions between the two compartments did not vary (Figure 2C), indicating that exploration was not affected by perinatal EtOH exposure.

#### OF

**Figure 3:**
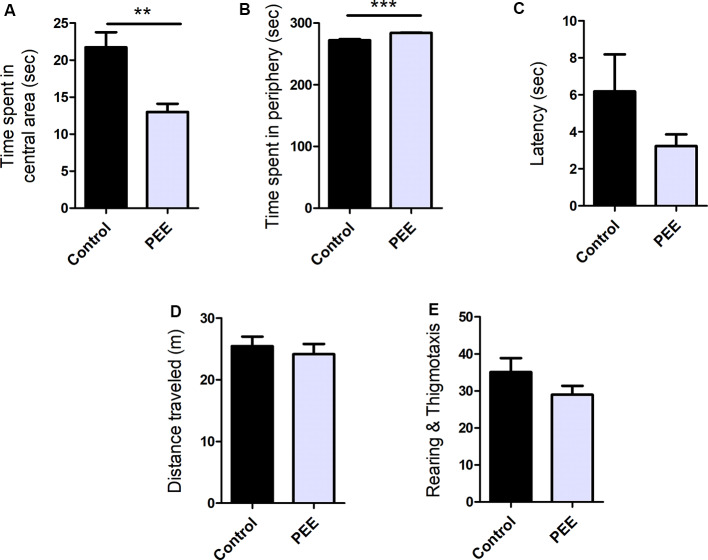
Perinatal EtOH exposure increases anxiety responses in the open field test (OF) test. Time spent in central area (in seconds, **A**), time spent in peripheral area (in seconds, **B**), latency to leave the center (in seconds, **C**), total distance traveled (in meters, **D**), number of rearings and thigmotaxis events **(E)**. Data expressed as the mean ± SEM; all parameters were analyzed by Student’s *t*-test (**p < 0.01, ***p < 0.001). Control *n* = 6; PEE *n* = 6, each data correspond to the average per litter.

PEE mice spent significantly less time in the central area (*t*_(10)_ = 3.784, *p* = 0.0036; Figure 3A) and more time in the periphery (*t*_(10)_ = 5.421, *p* = 0.0003; Figure 3B), and presented a tendency to exhibit shorter latency times than Control animals (Figure 3C). All these results are consistent with one another and indicate an anxiogenic effect of perinatal EtOH. The distance traveled (Figure 3D) and the number of rearings and thigmotaxis did not differ between groups (Figure 3E), which indicates unaltered horizontal and vertical locomotion in PEE animals and may reflect a certain specificity of the effect of EtOH on anxious behavior.

### Morphometric Parameters of ACC and Amygdala

Neither the organization of the six cortical layers of the ACC nor its thickness showed differences between PEE and Control groups (Supplementary Figure S4E). In both PEE animals and Controls, a radial organization of the cells of the cingulate cortex was observed towards the cingulum (Supplementary Figures S4A–D).

As shown in the histological analyses of the basolateral area, the amygdala cytoarchitecture was also conserved in PEE animals regarding the appearance observed in Controls (Supplementary Figures S5A–D). The area occupied by the amygdala in brain slices at the same Bregma level did not differ between the two groups (Supplementary Figure S5E).

**Figure 4:**
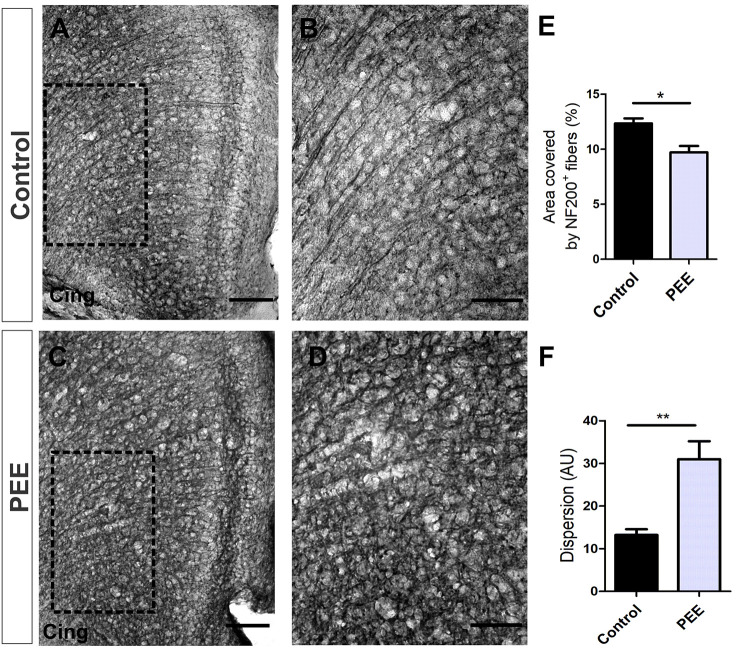
The axonal cytoskeleton is altered in adult PEE adults. Optical photomicrographs of coronal sections of adult male mouse brains immunostained with NF200. Height of the anterior cingulate cortex (ACC) of a Control **(A)** and PEE brain **(C)** at low magnification. Height of the ACC of a Control **(B)** and PEE brain **(D)** at higher magnification. In **(A,C)** photomicrographs, the cingulum is indicated with the abbreviation cing. Area covered by NF200^+^ fibers (%, **E**). Dispersion grade in the orientation of the NF200^+^ fibers is expressed in arbitrary units (AU; **F**). Data expressed as the mean ± SEM (Control *n* = 4 each one from four different control litters, PEE *n* = 4 each one from four different ethanol PEE litters); all parameters were analyzed by Student’s *t*-test (*p < 0.05, **p < 0.01). Scale bars: 100 μm **(A,C)**, 50 μm **(B,D)**.

The axonal cytoskeleton, immunolabeled for NF200 protein in ACC, was altered in adult PEE males (Figures 4A–D). Control animals showed axonal fibers with a radial distribution in this structure, while PEE animals exhibited disorganized axonal distribution through the six typical layers of the cerebral cortex (*t*_(6)_ = 4.054, *p* = 0.0067; Figure 4F) and smaller area covered (*t*_(6)_ = 3.582, *p* = 0.0116; Figure 4E).

**Figure 5:**
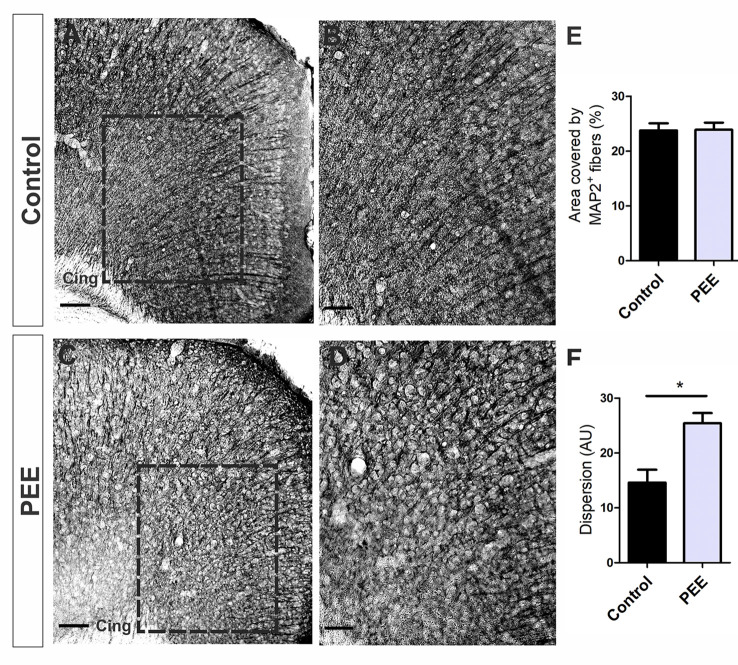
The dendritic cytoskeleton is altered in PEE adults. Optical photomicrographs of coronal sections of adult male mouse brains immunostained with MAP2. Height of the ACC of a Control **(A)** and PEE brain **(C)** at low magnification. Height of the ACC of a Control **(B)** and PEE brain **(D)** at higher magnification. Dispersion grade in the orientation of MAP2^+^ processes is expressed in arbitrary units (AU, **F**). In **(A,C)** photomicrographs, the cingulum is indicated with the abbreviation cing. Area covered by MAP2^+^ fibers (%, **E**). Data expressed as the mean ± SEM (Control *n* = 4 each one from four different control litters, PEE *n* = 4 each one from four different ethanol PEE litters); all parameters were analyzed by Student’s *t*-test (*p < 0.05). Scale bars: 100 μm **(A,C)**, 50 μm **(B,D)**.

Immunostaining for MAP2 protein (Figures 5A–D), which allows the identification of neurons and dendritic prolongation, also showed ACC radial organization in Controls but not in PEE animals (*t*_(6)_ = 3.612, *p* = 0.0112; Figure 5F), although the area covered by these fibers did not differ between groups (Figure 5E).

**Figure 6:**
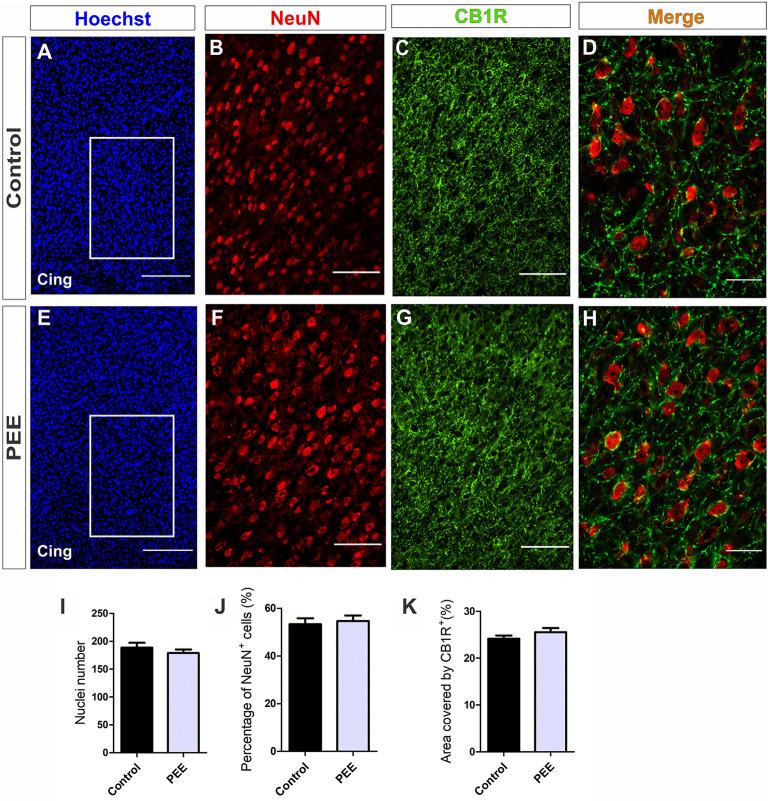
The population of mature neurons and the area covered by CB1 receptor (CB1R) are conserved in the anterior cingulate cortex (ACC) of PEE adults. Images of coronal sections of adult male mouse brains with Hoechst staining (blue) and immunofluorescence for NeuN (red) and CB1R (green) taken on an inverted microscope with spinning disk unit (SDU). Sections at the level of the ACC of a Control **(A)** and PEE brain **(E)** at low magnification. Sections at the level of the ACC of a Control **(B,C)** and PEE brain **(F,G)** at higher magnification. The merge of NeuN and CB1R immunofluorescence is shown at 60× magnification **(D,H)**. In **(A,D)** photomicrographs, the cingulum is indicated with the abbreviation cing. Nuclei number **(I)**, percentage of NeuN^+^ cells (%, **J**), area covered by CB1R (%, **K**). Data expressed as the mean ± SEM (Control *n* = 5 each one from five different control litters, PEE *n* = 5 each one from five different ethanol PEE litters); all parameters were analyzed by Student’s *t*-test. Scale bars: 200 μm **(A,E)**, 75 μm **(B,C,F,G)**, 25 μm **(D,H)**.

**Figure 7:**
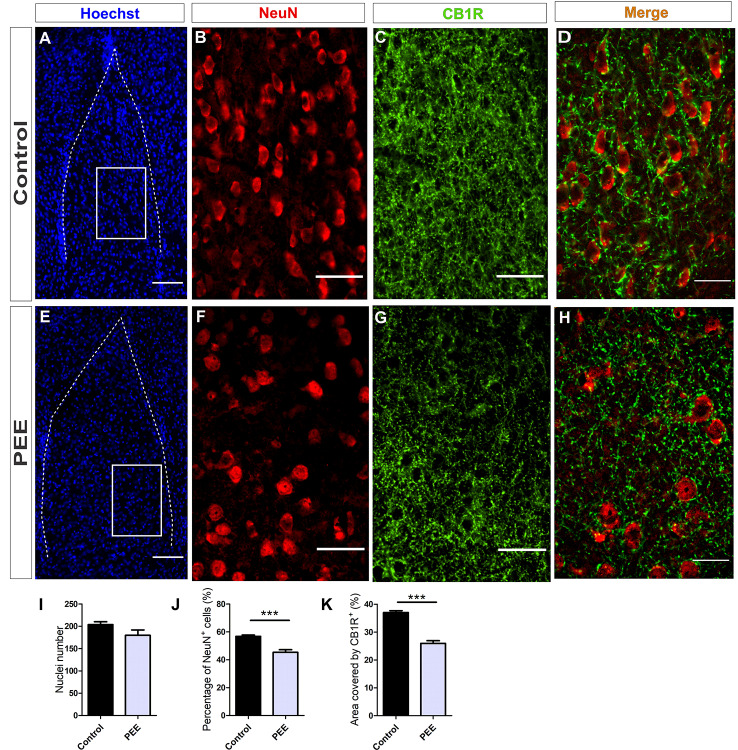
The population of mature neurons and the area covered by CB1R are smaller in the amygdala of PEE adults. Images of coronal sections of adult male mouse brains with Hoechst staining (blue) and immunofluorescence for NeuN (red) and CB1R (green) taken on an inverted microscope with SDU. Sections at the level of the amygdala of a Control **(A)** and PEE brain **(E)** at low magnification. Sections at the level of the amygdala of a Control **(B,C)** and PEE brain **(F,G)** at higher magnification. The merge of NeuN and CB1R immunofluorescence is shown at 60× magnification **(D,H)**. Nuclei number **(I)**, percentage of NeuN^+^ cells (%, **J**), area covered by CB1R (%, **K**). Data expressed as the mean ± SEM (Control *n* = 5 each one from five different control litters, PEE *n* = 5 each one from five different ethanol PEE litters); all parameters were analyzed by Student’s *t*-test (^***^p < 0.001). Scale bars: 100 μm **(A,E)**, 50 μm **(B,C,F,G)**, and 25 μm **(D,H)**.

Further histological analyses of the ACC (Figures 6A,E) regarding the cellularity and percentage of mature neurons (Figures 6B,F) showed no alterations in PEE animals (Figures 6I,J). In addition, the area covered by CB1R (Figures 7C,G) in this structure showed no significant differences between groups (Figures 6C,G,K).

At higher magnification, the expression of CB1R on the ACC is shown. CB1R is highly expressed around the mature neurons of the ACC in both Control and PEE brains (Figures 6D,H).

Studies conducted on the same parameters in amygdala (Figures 7A,E) revealed a conserved total cell number in PEE adults (Figure 7I) but a smaller percentage of mature neurons (Figures 7B,F,J) (*t*_(8)_ = 5.353, *p* = 0.0007) regarding Controls (Figures 7I,J). The area covered by CB1R (Figures 7C,G) in this structure was significantly smaller in PEE animals compared to Controls (*t*_(6)_ = 8.081, *p* = 0.0002; Figure 7K).

At higher magnification, the expression of CB1R on the amygdala is shown. CB1R is highly expressed around mature amygdala neurons in Control brains, but in PEE ones, this expression is reduced (Figures 7D,H).

**Figure 8:**
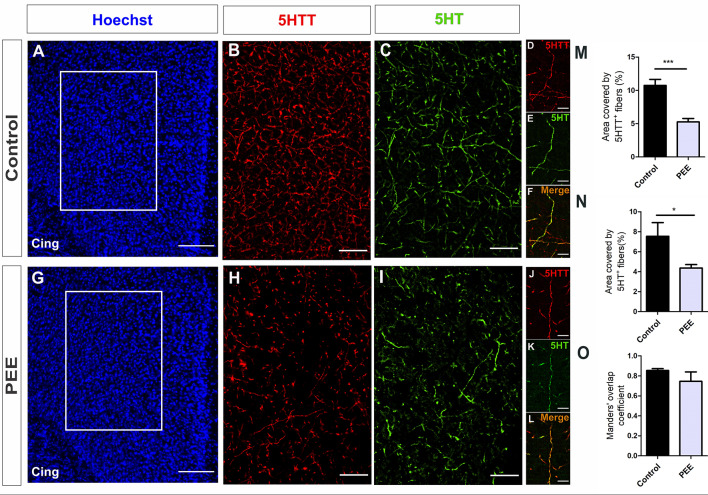
Serotoninergic innervation is altered in the ACC of adult PEE mice. Images of coronal sections of adult male mouse brains with Hoechst staining **(A,G)** and immunostained for 5HTT **(B,D,H,J)** and 5HT **(C,E,I,K)**. Sections at the level of the ACC of a Control **(A–F)** and PEE brain **(G–L)**. Merge images **(F,L)** show that fibers containing 5HTT also contain 5HT. In **(A,G)** photomicrographs, the cingulum is indicated with the abbreviation cing. Area covered by 5HTT^+^ fibers (%, **M**), area covered by 5HT+ (%, **N**), and Manders’ overlap coefficient **(O)**. Data expressed as the mean ± SEM (Control *n* = 4 each one from four different control litters, PEE *n* = 5 each one from five different PEE litters); all parameters were analyzed by Student’s *t*-test (*p < 0.05, ***p < 0.001). Scale bar: 150 μm **(A,G)**, 50 μm **(B,C,H,I)**, and 10 μm **(D–F,J–L)**.

**Figure 9:**
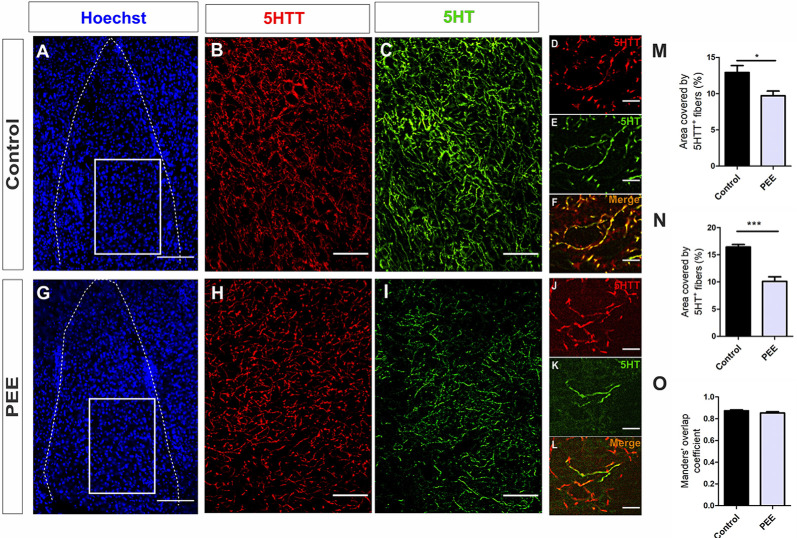
Serotoninergic innervation is altered in the amygdala of adult PEE mice. Images of coronal sections of adult male mouse brains with Hoechst staining **(A,G)** and immunostained for 5HTT **(B,D,H,J)** and 5HT **(C,E,I,K)**. Sections at the level of the amygdala of a Control **(A–F)** and PEE brain **(G–L)**. Merge images **(F,L)** show that fibers containing 5HTT also contain 5HT. In **(A,G)** photomicrographs, the cingulum is indicated with the abbreviation cing. Area covered by 5HTT^+^ fibers (%, **M**), area covered by 5HT+ (%, **N**), and Manders’ overlap coefficient **(O)**. Data expressed as the mean ± SEM (Control *n* = 4 each one from four different control litters, PEE *n* = 4 each one from four different PEE litters); all parameters were analyzed by Student’s *t*-test (*p < 0.05, ***p < 0.001). Scale bar: 150 μm **(A,G)**, 50 μm **(B,C,H,I)**, and 10 μm **(D–F,J–L)**.

Regarding serotonergic neuromodulation, PEE animals showed alterations in serotonergic afferences (Figures 8A–O and Figures 9A–O) as evidenced by a decrease in the area covered by fibers immunostained with the serotonin transporter in both the ACC (*t*_(7)_ = 5.564, *p* = 0.0008; Figure 8M) and amygdala (*t*_(6)_ = 2.861, *p* = 0.0287; Figure 9M). These 5HTT-positive fibers were also 5HT-positive, as shown in merge images of Figures 8F,L, 9F,L. The area covered by 5HT^+^ immunostaining was lower in PEE animals in both ACC (*t*_(7)_ = 2.518, *p* = 0.0399; Figure 8N) and amygdala (*t*_(6)_ = 6.613, *p* = 0.0006; Figure 9N). Taking both results, we can conclude that the serotonergic innervation was lower in PEE animals.

The Manders’ overlap coefficient mean value was near 0.8 in all cases and did not differ between the two groups (Figures 8O, 9O), which indicates that there is an approximately 80% of superposition of 5HTT^+^ and 5HT^+^ immunofluorescent structures and confirms that 5HTT^+^ fibers contain the neurotransmitter 5HT.

**Figure 10:**
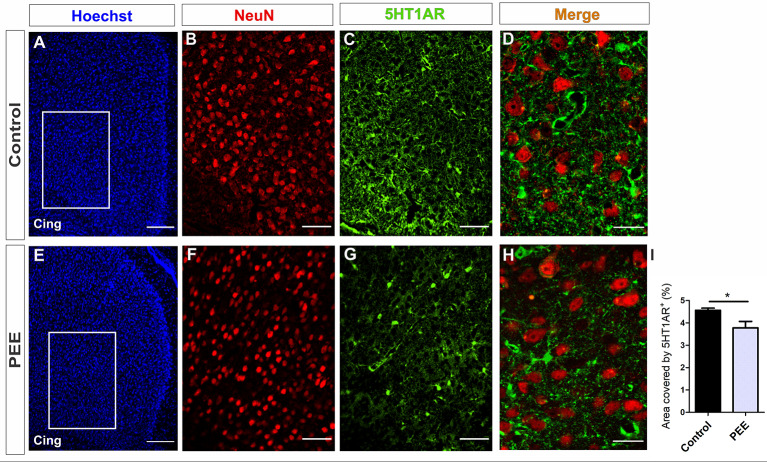
5HT1A receptor expression is altered in the ACC of PEE adults. Images of coronal sections of adult male mouse brains with Hoechst staining **(A,E)**, immunofluorescence for NeuN **(B,F)**, 5HT1A receptor (5HT1AR; **C,G**), and merge **(D,H)** taken on an inverted microscope with a SDU. Sections at the level of the ACC of a Control **(A–D)** and PEE brain **(E–H)**. In A and G photomicrographs, the cingulum is indicated with the abbreviation cing. Area covered by 5HT1AR^+^ measured in the fields delimited by boxes (%, **I**). Data expressed as the mean ± SEM (Control *n* = 5 each one from five different control litters, PEE *n* = 3 each one from three different ethanol PEE litters); all parameters were analyzed by Student’s *t*-test (*p < 0.05). Scale bar: 150 μm **(A,E)**, 50 μm **(B,C,F,G)**, and 20 μm **(D,H)**.

**Figure 11:**
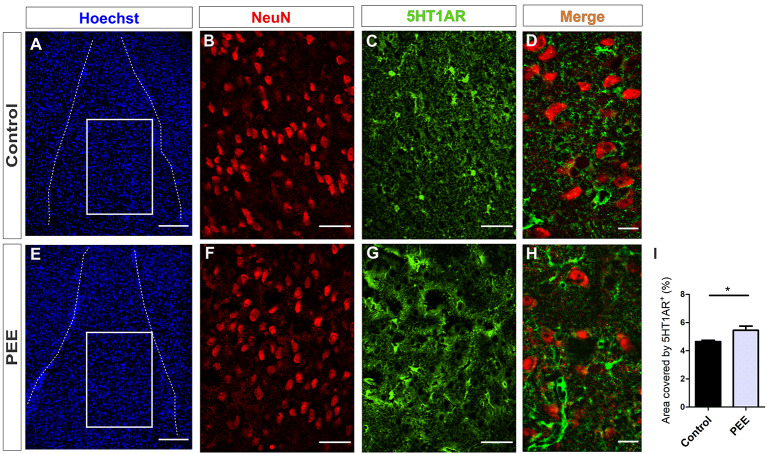
5HT1A receptor expression is altered in the amygdala of PEE adults. Images of coronal sections of adult male mouse brains with Hoechst staining **(A,E)**, immunofluorescence for NeuN **(B,F)**, 5HT1AR **(C,G)**, and merge **(D,H)** taken on an inverted microscope with a SDU. Sections at the level of the amygdala of a Control **(A–D)** and PEE brain **(E–H)**. Area covered by 5HT1AR^+^ measured in the fields delimited by boxes (%, **I**). Data expressed as the mean ± SEM (Control *n* = 4 each one from four different control litters, PEE *n* = 3 each one from three different ethanol PEE litters); all parameters were analyzed by Student’s *t*-test (*p < 0.05). Scale bar: 150 μm **(A,E)**, 50 μm **(B,C,F,G)**, and 15 μm **(D,H)**.

In addition, PEE males showed alterations in 5HT1A receptor levels regarding Controls (Figures 10A–I and Figures 11A–I) with a decrease in the ACC (*t*_(6)_ = 3.136, *p* = 0.0202; Figure 10I) and an increase in the amygdala (*t*_(5)_ = 2.943, *p* = 0.0321; Figure 11I).

At higher magnification, the expression of the 5HT1A receptor around the mature neurons of the ACC can be observed, which is lower in the ACC of PEE animals (Figures 10D,H). On the contrary, in the amygdala, the expression of this receptor around the mature neurons in the PEE animals is higher than the Controls (Figures 11D,H).

## Discussion

The experience and behavior that parents have had prior to conception can affect future offspring, as behavioral patterns such as diet (Öst et al., 2014), exercise (Denham, 2018) or drug exposure (Minnes et al., 2014) may generate epigenetic marks in individuals, such as DNA methylation. In this way, the offspring inherit not only the genes of the parents but also their previous experience translated into epigenetic marks. In the current study, the EtOH exposure animal model contemplates not only the direct impact of this drug on the gestation and lactation but also the effects of pregestational exposure, i.e., EtOH consumption by the dam prior to pairing. Our mouse model thus intends to reproduce the pattern of EtOH consumption of an alcoholic mother, taking into account experiences prior to the conception of the offspring.

One of the problems associated to the administration of EtOH into the beverage is that rodents tend to dislike it. Reports have shown that Wistar rats refuse to drink EtOH 10% v/v but are capable of drinking a solution of EtOH 6% v/v in water for 4 weeks, with no symptoms of toxic effect in hepatic tissue or alterations in their ability to mate, pregnancy parameters, lactation, or pup care, and yield moderate to low BEC values in both dams and pups (Evrard et al., 2003). In contrast, intraperitoneal administration through an injection of 3.5 g/kg/day to pregnant Wistar rats from G10 to G18 has rendered higher BEC and some teratogenic consequences (Aronne et al., 2008). In addition, pregnant Long Evans rats intraperitoneally injected with EtOH 2.9 g/kg on G15 and EtOH 1.45 g/kg 2 h later have shown BEC values of 287 ± 3.5 mg/dl (Mooney and Varlinskaya, 2011). Furthermore, pregnant CD1 females administered EtOH 25% v/v in the beverage have rendered pregnancy BEC values of 100–140 mg/dl (Kozanian et al., 2018).

The protocol used in this work shows that the BEC reached by E mothers was slightly higher than the legal limit for driving in Argentina (50 mg/dl) and below the levels of overt drunkenness in humans (200 mg/dl). In this way, considering that consequences of prenatal EtOH exposure depend on the dose, period, and duration of EtOH exposure (Petrelli et al., 2018), the current work contemplates the consequences that moderate and prolonged maternal consumption of EtOH may have on offspring and might be thus thought to mimic cases of FASD.

Previous studies by our group using administration of EtOH 6% v/v to Wistar rats through a liquid diet before and during gestation have shown dam BEC of 89.34 ± 6.42 mg/dl, as well as alterations in fetal brain morphology that affect the development of radial glia and hence cause a delay in migration. This could induce a disruption in the structure and function of major CNS laminated structures such as the cerebral cortex (Aronne et al., 2011). On the other hand, adolescent rats perinatally exposed to EtOH at this concentration show a higher preference for EtOH (more significant in females than males) and behavioral alterations (Aronne et al., 2013). In other words, prenatal or perinatal EtOH exposure in rodents produces changes in behavior that have been extensively studied from early postnatal days until early adulthood and has an impact on CNS structures that could be related to these alterations. Similar protocols of exposure to EtOH drinking have also been used in different mouse strains, some of them even involving a high EtOH concentration as the only beverage (Kleiber et al., 2011; El Shawa et al., 2013; Vega et al., 2013; Pérez-Tito et al., 2014; Abbott et al., 2016). At the moment, however, no conclusive results have been obtained regarding the behavior of adult male mice perinatally exposed to low/moderate concentrations of EtOH.

Anxiety is defined as a negative emotional state associated with the perception of potential or ambiguous threat. No unequivocal measures of anxiety have been yet established for rats and mice; however, and even when they may render differences between strains or face methodological criticism, the LDB and OF tests are generally accepted as a measurement of rodent anxious behavior, as they may assess fear-induced escape/avoidance or spontaneous natural preference for enclosed or unlit spaces (Ennaceur, 2014).

Previous studies in adult rodents prenatally exposed to EtOH showed similar results to those observed in this work, even using treatments applied in different time windows, with higher concentrations of EtOH and supplied through different routes (Hellemans et al., 2008; Kleiber et al., 2011; Cullen et al., 2013; Wieczorek et al., 2015). In addition, behavioral studies in rats prenatally exposed to EtOH between G17 and G20 have revealed an anxious phenotype in childhood and adolescence (Wille-Bille et al., 2018). In contrast, other authors have reported a decrease in anxiety-like behavior in adult and adolescent rodents prenatally exposed to EtOH, even using similar treatments and the same behavioral tests used in the current work (Osborn et al., 1998; Allan et al., 2003; Carneiro et al., 2005; Ohta et al., 2010; Diaz et al., 2016). Other groups have demonstrated that CD1 mice exposed to EtOH 25% v/v during gestation show an anxious phenotype at P20 and P50 (El Shawa et al., 2013; Abbott et al., 2016). In this work, adult male PEE mice of the CD1 strain exhibited an anxious phenotype that was consistent throughout the behavioral tests used. This phenotype is in agreement with that observed in humans, with studies showing that children, adolescents, and even adults prenatally exposed to EtOH present frequent psychiatric disorders such as anxiety (Famy et al., 1998; O’Connor and Paley, 2009; Popova et al., 2016; Weyrauch et al., 2017).

The formation of the complex architecture of the mammalian cerebral cortex requires orchestrated events including neural stem cell proliferation, migration, and neuronal differentiation. Successful neural migration involves three basic steps: initial departure of neuroblasts from the ventricular zone, migration to the cortical plate, and final settlement at their intrinsic laminar positions (Pang et al., 2008). Cortical connections formed during gestation and infancy are modified through synaptic pruning and cellular apoptosis. We have shown in previous work that the cerebral cortex of fetuses exposed to EtOH has a delay in neuroblast migration that produces alterations in lamination (Aronne et al., 2011) and that adult offspring prenatally exposed to EtOH have a thinner cerebral cortex, also with alterations in lamination (Aronne et al., 2013). These results confirm that exposure to EtOH during brain development produces morphological changes that persist into adulthood even in the absence of EtOH consumption.

Similar results, including low body and brain weights as well as lower cerebral cortex thickness, have been observed in infant and adult CD1 mice prenatally exposed to EtOH 25% v/v during gestation (El Shawa et al., 2013; Abbott et al., 2016). In addition, C57Bl/6 mice exposed to a liquid diet of EtOH 10% v/v from 15 days before pregnancy up to P4 revealed a reduction in olfactory bulb, hippocampus granule cell layer of the dentate gyrus, and fourth ventricle volume in adulthood, but larger amygdala volume (Akers et al., 2011).

Moreover, previous studies in which female mice were treated with EtOH 10% v/v prior to conception and during gestation and lactation have evidenced an anxiety-like behavior in youth offspring (Kleiber et al., 2011). On the other hand, Pascual et al. (2017) observe an anxiety-like behavior in adult PEE offspring exposing the dams to EtOH 2 months before conception until the end of lactation. In this work, authors also observed an increase in markers associated with inflammation processes in the brain that could be related with the neurodevelopmental defects registered (Pascual et al., 2017).

In turn, the brain region evaluated in this work is associated with cognitive processes (Kim et al., 2014; Meechan et al., 2015) and complex behavior such as response to fear and anxiety (Jhang et al., 2018; Sah et al., 2019). In particular, studies focused on areas of the limbic system like the cingulate cortex and amygdala, whose cytoarchitecture and state of synaptic connections may be linked to alterations in functionality and, ultimately, in the behavioral aspects they regulate. Therefore, the reduction observed in the population of mature neurons in the amygdala of PEE adult mice could be related to the anxious phenotype recorded in them. Similar results on the correspondence between morphology of the amygdala and behavior in adult CD1 mice prenatally exposed to EtOH have been obtained by other authors (Kozanian et al., 2018). Moreover, these results could be linked to clinical evidence showing that patients with autism have an increase in anxiety and a lower number of neurons in this structure (Schumann and Amaral, 2006).

NF200, a dynamic element of the neuronal cytoskeleton, determines axonal caliber and is necessary for axonal growth and guidance on their way to the synaptic target, as well as for neuronal shaping (Hoffman et al., 1987). We have observed a decrease in NF200 expression in the cingulate cortex of PEE adult mice that could be related to its functionality, either by an alteration in efferences, causing changes in the behavior it controls, or by an alteration in afferences, altering its regulation and, consequently, events downstream.

It is known that in mice prenatally exposed to EtOH, mesencephalic serotonin nuclei have a lower number of serotonergic neurons at P45 (Sari and Zhou, 2004) and a lower content of serotonin in the whole adult brain (Krsiak et al., 1977). In our work, both 5HTT and 5HT immunofluorescence were used to evaluate serotonergic innervation in the ACC and amygdala, showing a significant decrease in the area covered by these fibers in both brain areas in PEE adults. This result implies an alteration in innervation and serotonergic control in these areas as a consequence of EtOH exposure during early brain development and is consistent with other reports showing alterations in 5HTT and 5HT levels in PEE offspring (Zafar et al., 2000; Ramos et al., 2002; Evrard et al., 2003). A decrease in 5HTT and 5HT may indicate a reduction in serotonergic innervation due to altered development in 5HT fibers as a result of PEE. Since the serotonergic system is neuromodulatory, this decrease may imply alterations in the regulation of these areas of the limbic system. Also, a decrease was observed in 5HT1AR expression in ACC, which, together with the decrease in 5HTT levels in this area, could indicate a deficit in serotonergic modulation as a consequence of PEE. 5HT1AR acts during early postnatal development to establish normal anxiety-like behavior in adults (Gross et al., 2002). Given this evidence, it might be speculated that a decrease in the levels of 5HTT, 5HT, and 5HT1AR in the ACC due to exposure to EtOH during fetal and early postnatal development is related to the anxiety-like behavior expressed in adulthood. In amygdala, however, a reduction in serotonergic innervation was accomplished through an increase in the expression of the 5HT1AR, suggesting that this brain structure has a compensatory response to the low level of 5HT in adult PEE.

The endocannabinoid system constitutes another neuromodulator and is associated to the regulation of anxious responses (Navarro et al., 1993; Rodríguez de Fonseca et al., 1997), with some reports specifically linking the basolateral amygdala with this type of behavior (Delgado et al., 2006). It is well known that glutamatergic projections toward the ventral hippocampus give rise to anxious responses and that the inactivation of the amygdala blocks anxious behavior (Janak and Tye, 2015). In addition, previous studies have shown that CB1R in the basolateral amygdala is mainly located in the synaptic terminations of the GABAergic type (Katona et al., 2001). The cannabinoid system has a biphasic role in the control of anxiety, being located in both glutamatergic and GABAergic terminals, which exert their effects on anxiety in opposite ways (Millan, 2003). Therefore, this system may be thought to function as a “buffer,” regulating the release of these two neurotransmitters in relation to alterations in serotonergic modulation also recorded in this area. Finally, in the current work, the expression of CB1R was found to decrease in the basolateral amygdala in PEE animals, with no changes in the ACC. Therefore, the cannabinoid system may regulate the functionality of the amygdala, a key structure of the limbic system due to its relationship with other areas, even with the cingulate cortex.

Some authors have suggested a possible crosstalk between the serotoninergic and endocannabinoid systems, demonstrating the presence of CB1 receptors in serotoninergic neurons (Lau and Schloss, 2008). A colocalization of CB1 receptor in serotoninergic fibers has even been demonstrated in the amygdala (Ashton et al., 2006; Häring et al., 2007). Taking into account the results presented in this work, it could be speculated that, in part, the decrease in CB1 levels in the amygdala of PEE animals could lead to alterations in the serotoninergic neuromodulation of this structure, which could have an impact on anxious behavior.

## Conclusion

Exposure to low/moderate concentrations of EtOH from conception to childhood produces morphological changes in the brain that can be detected in adulthood even with no further EtOH consumption. In sum, some of the morphological alterations produced by EtOH are never reversed and remain in areas of the limbic system related to emotion where two of the main neuromodulatory systems, serotonergic and cannabinoid, also suffer alterations that might account for later anxious-like behavior.

## Data Availability Statement

The datasets generated for this study are available on request to the corresponding author.

## Ethics Statement

The animal study was reviewed and approved by CICUAL, Facultad de Medicina, Universidad de Buenos Aires, Res. 2375/2017.

## Author Contributions

CM conducted all the steps in the experimental procedures (EtOH administration, control of gestation and lactation, behavioral tests, and morphological studies), data processing, statistical analysis, and wrote an initial draft of the article. NV participated with CM in animal fixations, immunostaining, and photographs. DS designed and analyzed with CM the behavioral tests. AB designed the experimental model, supervised the course of experiments, and wrote the final version of the article. All authors revised the final version of the article.

## Conflict of Interest

The authors declare that the research was conducted in the absence of any commercial or financial relationships that could be construed as a potential conflict of interest.

## Footnotes

^1^http://imagej.net
