# Supplementary material for: Anxious Behavior of Adult CD1 Mice Perinatally Exposed to Low Concentrations of Ethanol Correlates With Morphological Changes in Cingulate Cortex and Amygdala
Source: Front Behav Neurosci. 2020 Jun 19;14:92. doi: 10.3389/fnbeh.2020.00092 (PMC7319189; doi:10.3389/fnbeh.2020.00092)
Supplement: Supplementary file 1 [file Table_1.docx]

**Supplementary data**

**1-Animal model**





**Supplementary figure 1**. Model parameters. (A) Ethanol consumption during all periods of treatment. Each bar represents the mean of ethanol intake expressed as g of ethanol consumed per gram of animal per day (g/g/day) ±SEM in the last week pregestational (n=6), in the 3 gestational weeks (n=6) and in the first week of lactation (n=5). (B) Beverage intake of the mothers during all periods of treatment. Each bar represents the mean of beverage intake expressed as ml of water in C or EtOH 6% v/v in E consumed per gram of animal per day (ml/g/day) ±SEM in the last week pregestational (C n=4, E n=4), in the 3 gestational weeks (C n=6, E n=6) and in the first week of lactation (C n=4, E n=4). (C) Mothers body weight gain during all the periods of treatment. Each bar represents the mean of the g of body weight gained from the beginning of each period (g) ±SEM in the last week pregestational (C n=4, E n=4), in the 3 gestational weeks (C n=6, E n=6) and in the first week of lactation (C n=4, E n=4). B and C Parameters were analyzed by two-way ANOVA test, were no significant differences were found between E and C groups.

| **Litter** | **Group** | **# Males LDB** | **# Males OF** |
| --- | --- | --- | --- |
| Control 1 | Control | 10 | 8 |
| Control 2 | Control | 6 | 6 |
| Control 3 | Control | 4 | 2 |
| Control 4 | Control | 2 | 3 |
| Control 5 | Control | 4 | 7 |
| Control 6 | Control | 4 | 6 |
| PEE 1 | PEE | 4 | 5 |
| PEE 2 | PEE | 5 | 5 |
| PEE 3 | PEE | 3 | 3 |
| PEE 4 | PEE | 3 | 5 |
| PEE 5 | PEE | 2 | 4 |
| PEE 6 | PEE | 5 | 6 |

**Supplementary Table 1**. Litter representation in behavioral tests. Number of males per litter in each behavioral test used to calculate averages.

**2- Negative control of immunostaining**

**
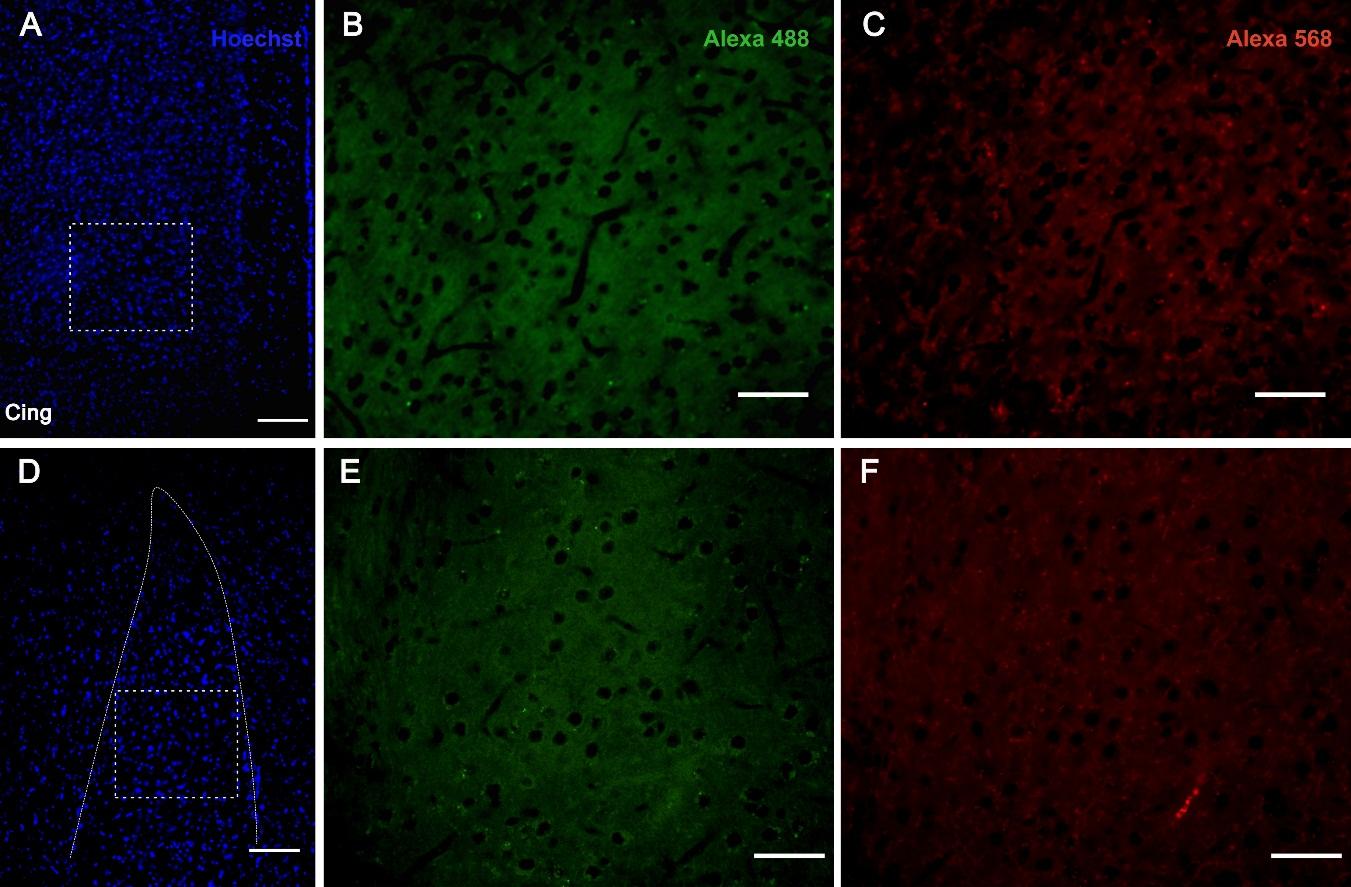
**

**Supplementary figure 2.** Control of immunofluorescence background (negative controls). Sections of the ACC (upper panel) and amygdala (lower panel) were incubated with fluorescent secondary anti-rabbit and anti-mouse antibodies conjugated with Alexa Fluor^TM^ 488 (B and E) and Alexa Fluor^TM^ 568 (C and F), respectively, and counter-dyed with Hoechst 33342 (A and D). Scale bar: 100 µm (A and D) and 50 µm (B, C, E and F).

**3- Quantification of fiber directionality**

3.1**-** Thresholding and binarization: Before morphometric analysis of fiber directionality, it is necessary to select pixels representing only the structures to be analyzed. Data processing results in a binary image in which pixels assigned to the selected structures take a value of 1 and pixels in the background take a value of 0.

ImageJ software processes the following algorithm: *Image/Adjust/Threshold/Apply*, which divides the image into “objects” and “background”.

3.2- The images are 700 x 300 µm and were taken in approximately the same layer of the ACC as shown in Supplementary figure 3. The images were processed to highlight the structure to be analyzed (subtract the background, enhance contrast).

The Directionality plugin (https://imagej.net/Directionality) created by Jean-Yves Tinevez (http://pacific.mpi-cbg.de/wiki/index.php/Directionality) was used.

The Fourier power spectra of each input image were analyzed using spatial filters. The statistical analysis selected the highest peak in each area, with 'Direction' reporting the center of a peak-adjusted Gaussian function, 'Dispersion' reporting the standard deviation of that peak-adjusted Gaussian function, and 'Amount' giving the sum of the values of the histogram from center ± SD divided by the total sum of the values of the histogram. The gradient of the image was calculated using a 5x5 Sobel filter and used to derive the gradient orientation of the fibers in a given area. The resulting histogram shows the preferential orientation, both quantifying it and indicating the degree of dispersion of that orientation in the 2D image.


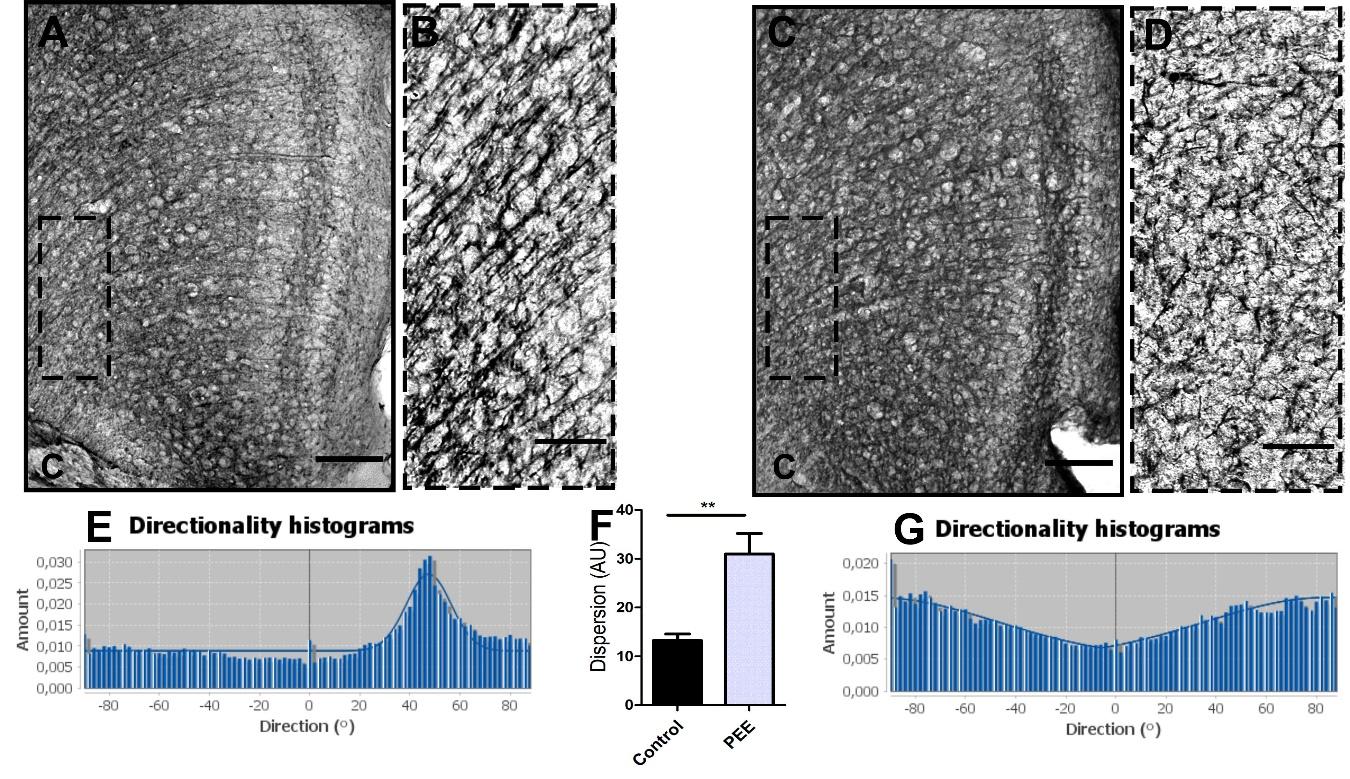


**Supplementary figure 3.1.** Analysis of N200+ fibers organization thought the ACC layers in control and PEE adult mice. Immunostaining sections of ACC of a Control (A) and PEE brain (C), the dotted line rectangle show the area analyzed at higher magnification (B and D). In A and C photomicrographs the cingulum is indicated with the abbreviation “C”. Histogram of the analysis of the N200+ fibers orientation on ACC of a Control (E) and PEE brain (G) obtained with the plugin. Dispersion of the preferential orientation in both Control and PEE (AU, F). Data expressed as the mean ± SEM (Control n = 4, PEE n =4); all parameters were analyzed by Student's t-test (** p< 0.01).

Scale bars: 100µm (A and C), 50 µm (B and D).


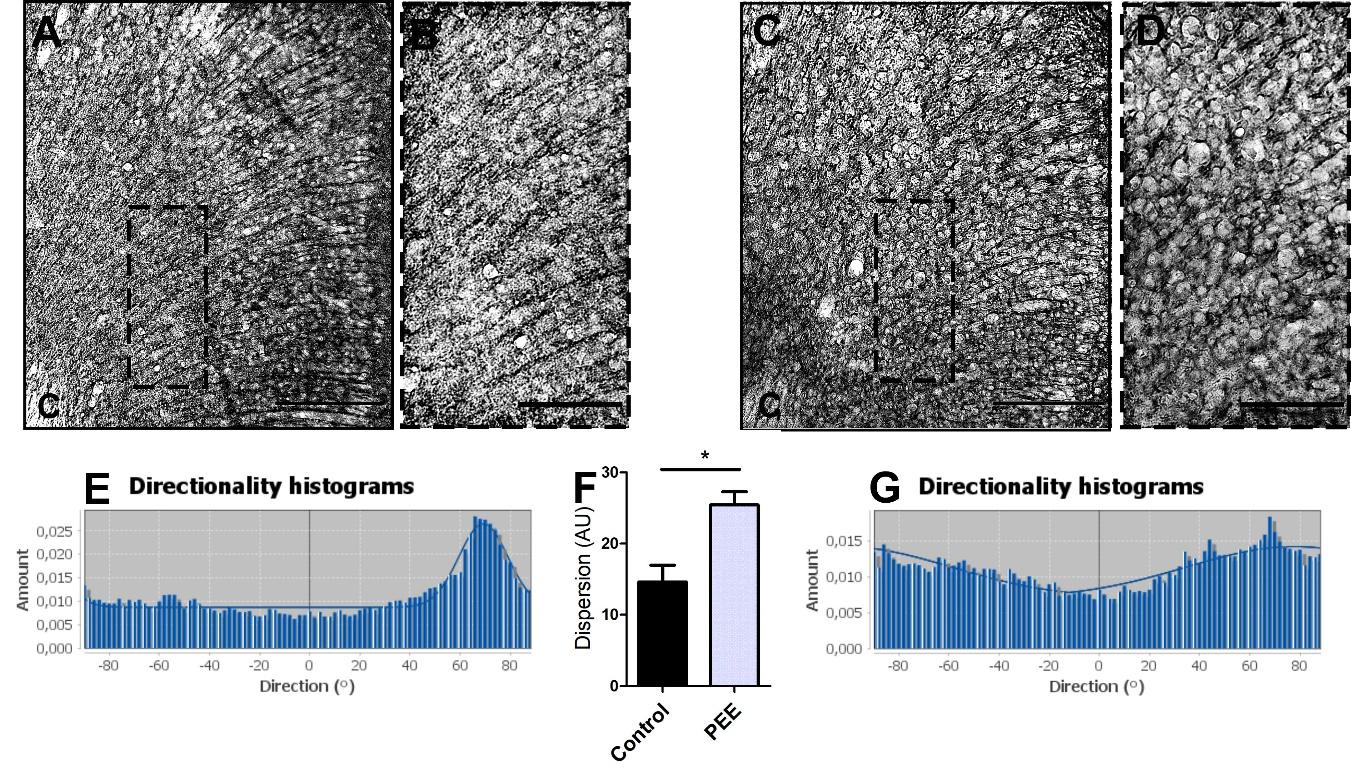


**Supplementary figure 3.2.** Analysis of MAP2+ fibers organization thought the ACC layers in control and PEE adult mice. Immunostaining sections of ACC of a Control (A) and PEE brain (C), the dotted line rectangle show the area analyzed at higher magnification (B and D). In A and C photomicrographs the cingulum is indicated with the abbreviation “C”. Histogram of the analysis of the MAP2+ fibers orientation on ACC of a Control (E) and PEE brain (G) obtained with the plugin. Dispersion of the preferential orientation in both Control and PEE (AU, F). Data expressed as the mean ± SEM (Control n = 4, PEE n =4); all parameters were analyzed by Student's t-test (*p< 0.05).

Scale bars: 100µm (A and C), 50 µm (B and D).

**4- Toluidine blue staining**

Five coronal 20-μm-thick brain sections containing ACC and amygdala were randomly selected from five different brains, each one from different litters, per experimental group. Slides were left at room temperature and then drops of Toluidine blue 0.5% (w/v) dissolved in an aqueous solution of sodium carbonate 2.5% (w/v) were spread on the tissue for 30 seconds. The solution was washed with distilled water and then with EtOH 96% (v/v). The slides were dried for 24 hours, dehydrated and coverslipped using Canada Synthetic Balm as mounting media. Photographs were taken in a Zeiss Axiolab microscope with several objectives (2,5X, 10X, 20X). Images were acquired using a CCD Q-Color 3 camera (Olympus) and QCapture 6.0 software.

**5- Morphometric digital images from Toluidine blue staining analysis**

From the Toluidine blue staining, the amygdala was delimited, and its area was measured with a digital tool (Region Of Interest, ROI); ACC thickness was measured by means of the length of the segment drawn between the inner pial and the cingulum. Both area and thickness were expressed in arbitrary units (AU), AU^2^ for area and AU for thickness.


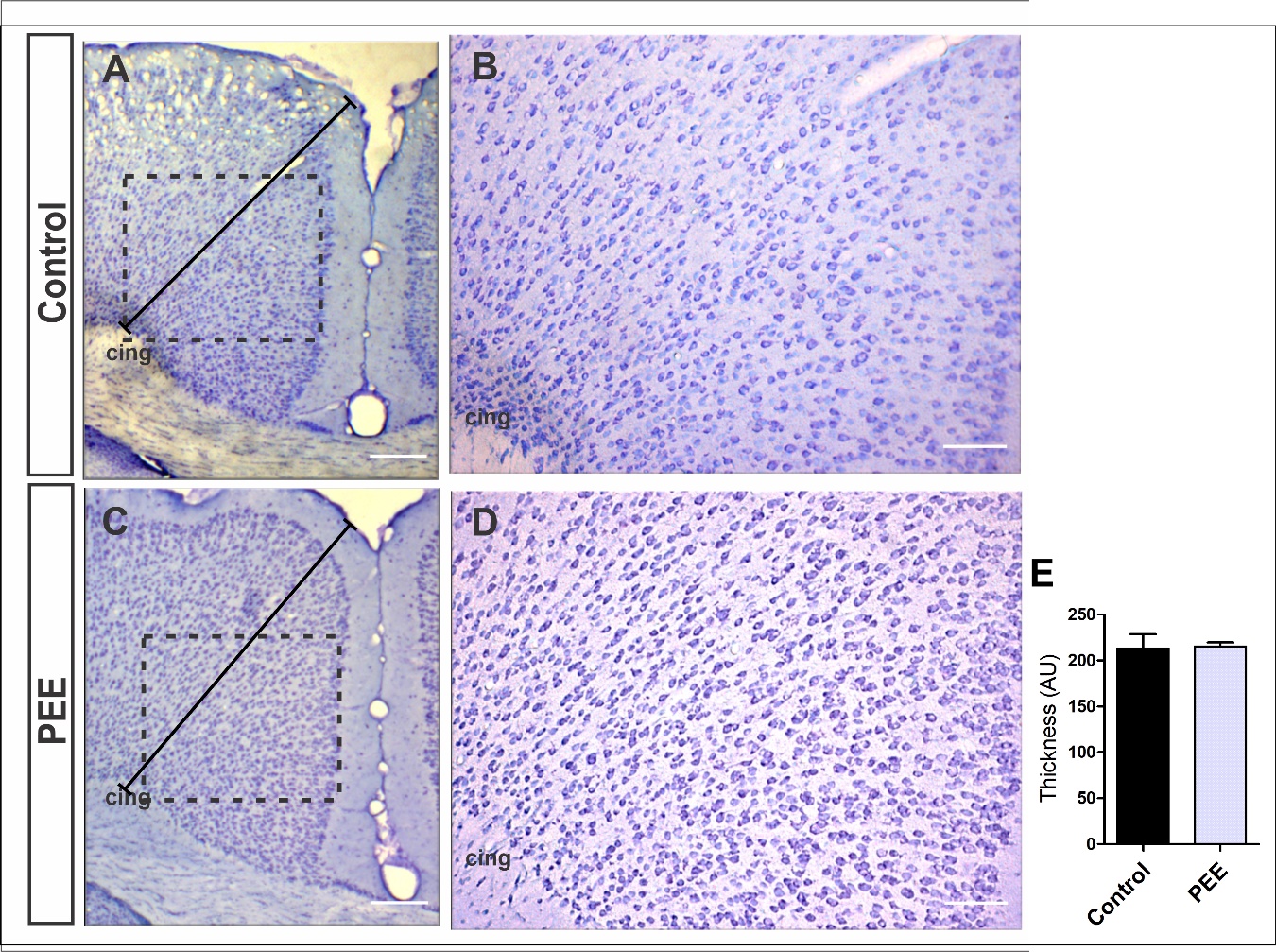


**Figure 4**. The histological structure of the ACC is conserved in PEE adults. Optical photomicrographs of coronal sections of adult male mouse brains stained with Toluidine blue. ACC of a Control (A) and PEE brain (C) at low magnification. In A and C, the segments represent the thickness of the ACC. ACC of a Control (B) and PEE brain (D) at higher magnification. In all photomicrographs the cingulum is indicated with the abbreviation cing. ACC thickness (E) is represented by arbitrary units (AU). Data expressed as the mean ± SEM (Control n = 5 each one from 5 different control litters, PEE n = 5, each one from 5 different ethanol litters); all parameters were analyzed by Student’s *t* test. Scale bars: 200 µm (A and C), 100 µm (B and D).


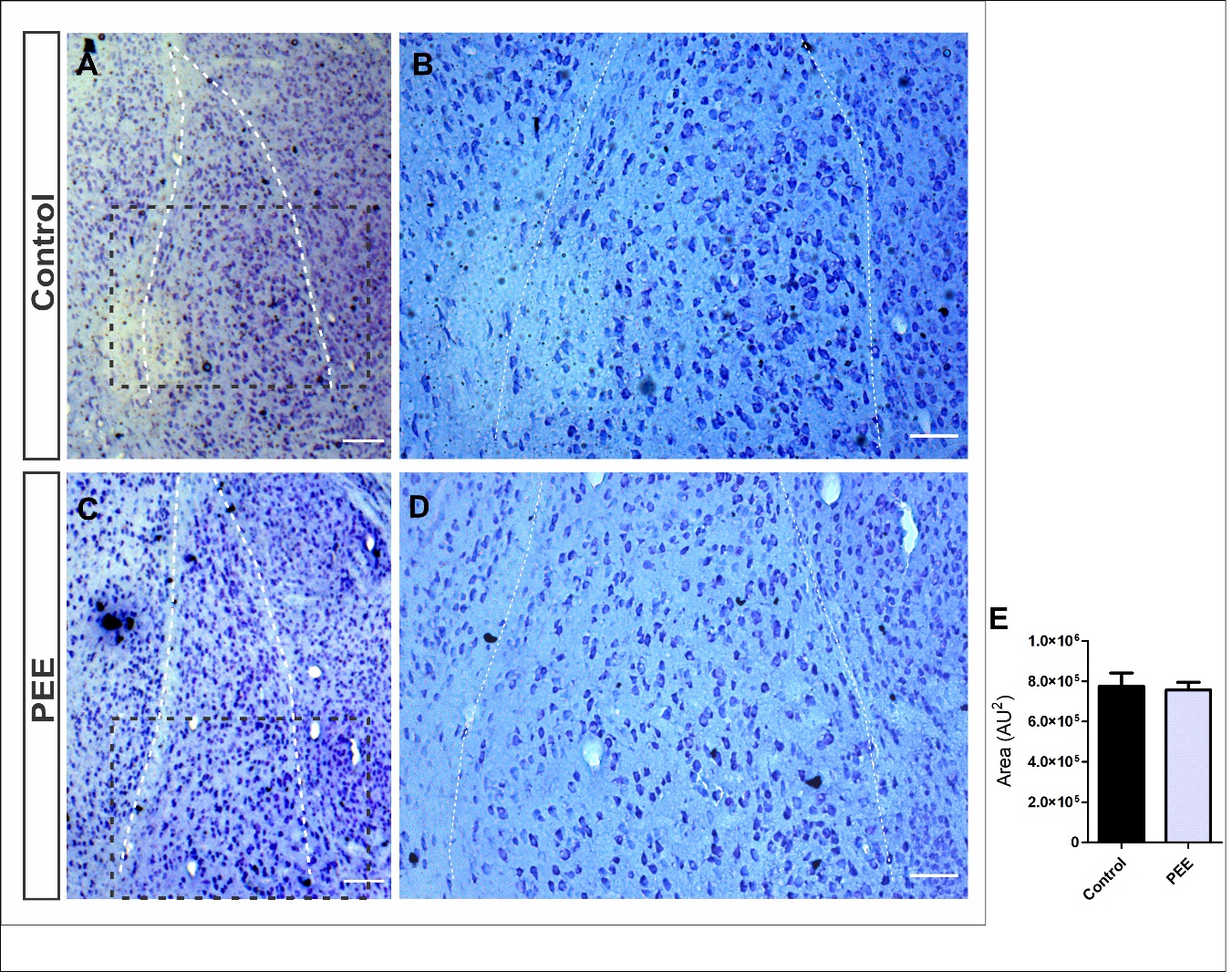


**Figure 5**. The histological structure of the amygdala is conserved in PEE adults. Optical photomicrographs of coronal sections of adult male mouse brains stained with Toluidine blue. Amygdala of a Control (A) and PEE brain (C) at low magnification. In A and C, the white dotted lines represent the borderline of the amygdala used to measure area. Amygdala of a Control (B) and PEE brain (D) at higher magnification, showing cellular distribution and organization. Amygdala area (E) is represented by arbitrary units squared (AU^2^). Data expressed as the mean ± SEM (Control n = 4 each one from 4 different control litters, PEE n = 5 each one from 5 different ethanol litters); all parameters were analyzed by Student’s *t* test. Scale bars: 200 µm (A and C), 30 µm (B and D).
